# Reward prediction-related increases and decreases in tonic neuronal activity of the pedunculopontine tegmental nucleus

**DOI:** 10.3389/fnint.2013.00036

**Published:** 2013-05-14

**Authors:** Ken-ichi Okada, Yasushi Kobayashi

**Affiliations:** ^1^Graduate School of Frontier Biosciences, Osaka UniversityOsaka, Japan; ^2^Center for Information and Neural Networks, National Institute of Information and Communications Technology, and Osaka UniversityOsaka, Japan; ^3^Research Center for Behavioral Economics, Osaka UniversityOsaka, Japan; ^4^Precursory Research for Embryonic Science and Technology, Japan Science and Technology AgencySaitama, Japan

**Keywords:** acetylcholine, reinforcement learning, pedunculopontine tegmental nucleus, reward prediction, cholinergic, tonic activity, motivation

## Abstract

The neuromodulators serotonin, acetylcholine, and dopamine have been proposed to play important roles in the execution of movement, control of several forms of attentional behavior, and reinforcement learning. While the response pattern of midbrain dopaminergic neurons and its specific role in reinforcement learning have been revealed, the roles of the other neuromodulators remain elusive. Reportedly, neurons in the dorsal raphe nucleus, one major source of serotonin, continually track the state of expectation of future rewards by showing a correlated response to the start of a behavioral task, reward cue presentation, and reward delivery. Here, we show that neurons in the pedunculopontine tegmental nucleus (PPTN), one major source of acetylcholine, showed similar encoding of the expectation of future rewards by a systematic increase or decrease in tonic activity. We recorded and analyzed PPTN neuronal activity in monkeys during a reward conditioned visually guided saccade task. The firing patterns of many PPTN neurons were tonically increased or decreased throughout the task period. The tonic activity pattern of neurons was correlated with their encoding of the predicted reward value; neurons exhibiting an increase or decrease in tonic activity showed higher or lower activity in the large reward-predicted trials, respectively. Tonic activity and reward-related modulation ended around the time of reward delivery. Additionally, some tonic changes in activity started prior to the appearance of the initial stimulus, and were related to the anticipatory fixational behavior. A partially overlapping population of neurons showed both the initial anticipatory response and subsequent predicted reward value-dependent activity modulation by their systematic increase or decrease of tonic activity. These bi-directional reward- and anticipatory behavior-related modulation patterns are suitable for the presumed role of the PPTN in reward processing and motivational control.

## INTRODUCTION

The pedunculopontine tegmental nucleus (PPTN) is the major source of cholinergic projections in the midbrain, but also contains glutamatergic, gamma aminobutyric acid (GABA)ergic, dopaminergic, and noradrenergic neurons (Mesulam et al., 1983; Rye et al., 1987; Clements and Grant, 1990; Jones, 1991; Spann and Grofova, 1992; Ford et al., 1995; Takakusaki et al., 1996; Wang and Morales, 2009). The PPTN controls sleeping/waking (Datta and Siwek, 2002; Kayama and Koyama, 2003) and locomotion (Garcia-Rill and Skinner, 1988; Takakusaki et al., 2004; Harris-Warrick, 2011), and also has a role in regulating motivated behavior (Rompre and Miliaressis, 1985; Kozak et al., 2005; Doya, 2008; Winn, 2008; Wilson et al., 2009; Okada et al., 2011). However, the role of the PPTN in motivated behavioral control remains rather elusive. On the other hand, there are numerous studies showing that another neuromodulation system, i.e., dopaminergic neurons located in the substantia nigra pars compacta and ventral tegmental area, play an essential role in the regulation of motivated behavior by encoding a reward prediction error signal for reinforcement learning (Schultz, 1998; Bromberg-Martin et al., 2010b). Dopaminergic neurons exhibit phasic burst firing in response to external stimuli and rewards, and their response magnitude alters throughout the course of learning to match the reward prediction error signal (Hollerman and Schultz, 1998). The PPTN projects to dopaminergic neurons (Beninato and Spencer, 1987), and these excitatory cholinergic/glutamatergic projections are thought to regulate the firing of dopaminergic neurons (Lokwan et al., 1999; Forster and Blaha, 2003; Mena-Segovia et al., 2008b). Thus, it is possible that neurons in the PPTN encode the reward-related signals that are necessary for the computation of the reward prediction error signal by dopaminergic neurons.

Many previous studies, including ours, reported the phasic activity of PPTN neurons in response to reward, sensory stimulus, and movement (Garcia-Rill and Skinner, 1988; Matsumura et al., 1997; Dormont et al., 1998; Kobayashi et al., 2002; Pan and Hyland, 2005; Okada and Kobayashi, 2009; Okada et al., 2009; Norton et al., 2011). Some studies also showed tonic changes in activity in relation to locomotion (Garcia-Rill and Skinner, 1988), sleeping/waking (Datta and Siwek, 2002), and arousal state (Mena-Segovia et al., 2008a). Previously, we recorded tonic changes in neuronal activity in the monkey PPTN while they performed a reward-biased saccade task, which was comparable to those used in recordings from dopaminergic neurons. We found that one group of PPTN neurons showed a tonic increase in activity during the task execution period, with greater activity during successful versus failed trials (Kobayashi et al., 2002) and greater activity during highly motivated trials (Okada et al., 2009). These neurons could act as a gate from motivation to action by changing attentional or arousal processes that matches the presumed role of the PPTN as the ascending reticular activating system (Steriade, 1996). Furthermore, some tonic excitatory neurons showed stronger responses to large reward-predicted cues than that to small reward-predicted cues (Okada et al., 2009). This group of PPTN neurons may provide the neural substrates for the temporal memory of the predicted reward magnitude, which is required for the computation of the reward prediction error.

Recent neurophysiological studies of the serotonergic dorsal raphe nucleus (DRN) reported that DRN neurons showed either an increase or decrease in tonic activity to reward-related cues and reward outcomes (Nakamura et al., 2008), and these tonic changes in their activity continually encode the state of expectation of future rewards, such that the response of a neuron to the start of the task was correlated with its response to the reward cues and outcomes (Bromberg-Martin et al., 2010a). The PPTN and DRN are interconnected with each other (Steininger et al., 1992; Honda and Semba, 1994), and serotonergic and cholinergic neuromodulatory systems control many brain functions, such as sleeping/waking (Kayama and Koyama, 2003) and locomotion (Takakusaki et al., 2004; Harris-Warrick, 2011), in a mutually interacting manner. Thus, similar bi-directional reward value coding might also be present in PPTN neurons.

To investigate the relationship between task-related tonic activity and reward-related modulation, we analyzed PPTN neuronal activity during several phases of a behavioral task, i.e., just before the start of the task, just after the start of the task, at reward cue presentation, and at reward delivery. In addition to the neurons we reported previously that exhibited an increase in tonic activity, other PPTN neurons showed a decrease in tonic activity during the behavioral task, similar to the activity of some DRN neurons. We found a correlation between the tonic increase or decrease in activity to the start of the task and the responses to large/small reward cues, but not to the delivery of large/small rewards. Furthermore, a partially overlapping population of neurons showed preparatory activity modulation that depended on the anticipatory fixational behavior of the monkeys. This result suggests that PPTN neurons encode both the externally cued reward value and internal anticipatory state signals by systematic changes in their tonic firing rate, both in excitatory and suppressive directions.

## MATERIALS AND METHODS

### GENERAL

We recorded neuronal activity from the PPTN in three Japanese macaque monkeys (*Macaca fuscata*; animal Ds, male; animal Tm, female; animal Dn, male) while they performed a reward-biased visually guided saccade task. All experimental procedures were performed in accordance with the National Institutes of Health *Guidelines for the Care and Use of Laboratory Animals* and approved by the Committee for Animal Experiments at Okazaki National Research Institutes and Osaka University.

Information on the experimental procedures was published previously (Kobayashi et al., 2002). Briefly, a head-holding device, a chamber for unit recording, and a scleral search coil were implanted under general anesthesia. During the experimental sessions, the animals were seated in a primate chair and placed in a sound-attenuated room. All aspects of the behavioral experiment, including presentation of the stimuli, monitoring of eye movements, monitoring of neuronal activity, and reward delivery, were under the control of a personal computer-based real-time data acquisition system (TEMPO) with a real-time link to MATLAB. Eye position was monitored by means of a scleral search coil system with a spatial resolution of 0.1° and time resolution of 1 kHz. The stimuli were presented on the screen of a 21-inch cathode ray tube monitor that was placed 28 cm in front of the animals.

### BEHAVIORAL TASK

**FIGURE 1:**
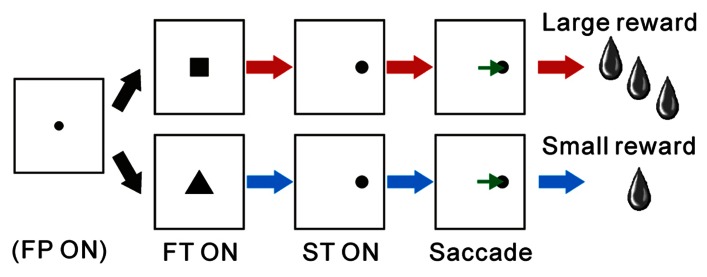
**Schematic diagram for the reward-biased visually guided saccade task**. The monkeys initially fixated on the central visual stimulus, then made a saccade to the peripheral target, and finally received a juice reward. During the fixation period before the visually guided saccade and reward delivery, the shape of the FT cued the animal to expect either a large or small reward for successful completion of the trial. In the recordings from animal Ds, an uninformative small FP was shown before FT presentation. FP, fixation point; FT, fixation target; ST, saccade target.

The animals performed a reward-biased visually guided saccade task. This task was comparable to those used in recordings from basal ganglia nuclei and dopaminergic neurons in which the shape of the fixation target (FT; square, circle, or triangle) indicated the reward magnitude (large or small, **Figure 1**). The monkeys initially fixated on the central target, then made a saccade to the peripheral target, and finally received a juice reward. During the initial fixation period, the shape of the FT cued the animals to expect either a large or small reward upon the successful completion of the trial. In the recordings from animal Ds, an uninformative small fixation point (FP) was presented initially at the center of the screen. The monkey was required to fixate on the FP within 3000 ms to a precision of ± 2°. After 400–800 ms fixation, the FP was replaced by a square or triangle FT, which was associated with the reward magnitude. In the recordings from animals Tm and Dn, the initial stimulus was a square or circle FT, and its shape was associated with large or small rewards, respectively. The FT shape-reward magnitude contingency was switched at quasi-random intervals (20–30 trials).

The subsequent task procedures were the same for all monkeys. After fixation on the FT for a variable duration (400–1500 ms), a saccade target (ST, a circle of 0.8°) appeared at an eccentricity of 10° from the FT in 1 of 2 (left or right) or 8 (0, 45, 90, 135, 180, 225, 270, and 315°) possible directions. The monkey was required to saccade to the ST within 80–500 ms to a precision of ±2°. Successful trials were rewarded with juice presented together with a tone at 100 or 300 ms after the ST disappeared. The large and small rewards consisted of 3 or 1 drops of juice (each drop ~0.1 ml), respectively. If the animal broke fixation at any time during the fixation period or failed to make a saccade to the ST, an error tone sounded and the trial was aborted. The intertrial interval, which started at the time of reward offset and lasted until the onset of the initial stimulus in the next trial, was fixed at 1500 ms in the recordings from animal Ds, and quasi-randomly varied (within 1.5–2 s) in the recordings from animals Tm and Dn.

### RECORDING PROCEDURE

Guide tubes held within the recording chamber were aimed at the PPTN of the monkeys using magnetic resonance imaging (2.2 T) under general anesthesia. The locations of the recorded neurons were reconstructed for two monkeys from the readings of the micromanipulator and those of the guide grids of the recording chamber, referenced to a single marker site selected for each monkey (Okada et al., 2009). Correct placement of the recording electrode was confirmed by monitoring the neuronal activity in the surrounding structures, including the auditory responses in the inferior colliculus encountered at 3–7 mm before those in the PPTN and high-frequency tonic fiber activity in the cerebellar peduncle, close to the PPTN.

While the PPTN is the major source of cholinergic projections in the brainstem (Mesulam et al., 1983), it also contains glutamatergic and GABAergic (Clements and Grant, 1990; Spann and Grofova, 1992; Ford et al., 1995; Takakusaki et al., 1996; Wang and Morales, 2009) as well as dopaminergic (Rye et al., 1987) and noradrenergic (Jones, 1991) neurons. It was suggested that there are two types of neurons that generate broad and brief action potentials, respectively, in slice preparations of the rat PPTN (Takakusaki et al., 1997). Recent extracellular recording studies also reported neurons that generated broad and brief action potentials; however, they exhibited a unimodal distribution and could not be classified into groups (Matsumura et al., 1997; Kobayashi et al., 2002). Therefore, rather than choosing neurons with specific electrophysiological properties, we studied all well isolated neurons in the PPTN whose activity changed during the saccade task.

### DATA ANALYSIS

Our database consisted of 507 neurons in animal Ds (saccade task with FP presentation), 156 neurons in animal Tm, and 29 neurons in animal Dn. The data from monkeys Tm and Dn are from the same neurons recorded in a previous study (Okada et al., 2009). The analysis included neuronal data from all correctly performed trials, excluding the first three trials of each block when the animals were adapting to the change in the FT shape-reward magnitude contingency.

We used receiver operating characteristic (ROC) analysis to compare the firing rates in two time windows during a trial or for two different task conditions. In principle, ROC analysis evaluates the reliability by which an ideal observer could correctly distinguish between 2 conditions from the neuronal signal. The ROC value is calculated as the probability that a randomly chosen firing rate from the first condition has a higher value than a randomly chosen firing rate from the second condition (excluding ties; Green and Swets, 1966). Thus, an ROC value 1 implies that neuronal activity in the first condition is always higher than in the second condition. An ROC value 0.5 implies that neuronal activity does not discriminate between the two conditions, and an ROC value zero implies that neuronal activity is always higher in the second condition.

For the analysis of task-related tonic changes in activity (see **Figure 4**), the normalized activity of each neuron was calculated as the ROC value comparing the firing rate of the neuron collected in a 200 ms window centered on that time versus the firing rate collected during a pre-fixation period represented by a 600 ms window before the onset of the initial stimulus to display neuronal activity during both the task period and intertrial interval. Neurons were classified as “tonic excitatory” or “tonic suppressive” based on their significant increase or decrease in activity during the post-fixation period (0–600 ms after the onset of the initial stimulus) versus their activity in the pre-fixation period (*p* < 0.05, Wilcoxon rank-sum test). We defined the fixation period response as the ROC value comparing the firing rate in the same 600 ms time window. An ROC value >0.5 implies that neuronal activity is increased after the onset of the initial stimulus.

For the analysis of reward-related modulation (see **Figure 5**), the normalized activity of each neuron was calculated as an ROC value separately for the large and small reward trials. We calculated reward-related modulation by comparing the firing rate in the large versus small reward trials, separately for the reward cue period (0–600 ms after FT onset) and outcome period (0–600 ms after reward delivery), using the Wilcoxon rank-sum test (*p* < 0.05) and ROC analysis. An ROC value >0.5 implies that neuronal activity is higher in the large reward condition (positive reward modulation). We also determined the contributions of the predicted reward magnitude and FT shape to the neuronal responses. Multiple linear regression analysis was performed in which the responses were modeled by a linear sum of the predicted reward magnitude and FT shape. From the regression coefficients of the model, we confirmed that the neuronal activity was significantly modulated by the predicted reward magnitude (Okada et al., 2009).

For the analysis of anticipatory behavior-related modulation (see **Figure 7**), we used the reaction time to fixate on the initial target (RTit) as a measure of the monkeys’ anticipation of the occurrence of an upcoming event. Even before the appearance of the initial stimulus, our monkeys frequently shifted their gaze to the center of the screen (entered a 3° window), i.e., they performed self-initiated movements based on their anticipation of an upcoming visual event. An RTit <0 implies that the monkey made an anticipatory gaze shift before the appearance of the initial target, while an RTit >0 implies that the monkey made a gaze shift after the appearance of the target. We classified the trials into short and long RTit categories (shorter and longer RTit than the median values of the individual neurons, respectively), and the normalized activity of each neuron was calculated as an ROC value separately for the short and long RTit trials. We calculated behavior-related modulation by comparing the firing rate in the short versus long RTit trials for the pre-fixation period (0–600 ms before the appearance of the initial stimulus) using the Wilcoxon rank-sum test (*p* < 0.05) and ROC analysis. An ROC value >0.5 implies that neuronal activity is higher in the short RTit condition (positive behavioral modulation).

Correlations between the fixation period response, reward-related modulation, and behavior-related modulation were assessed using Spearman’s rank correlation. To estimate the significance of the correlation, we performed a permutation test by shuffling each dataset 20,000 times.

The electrophysiological properties of neurons were quantified, such as spike duration, spiking irregularity, and firing rate. Spike duration was measured between the first negative deflection and the peak of the second positive deflection of the spike waveform. Spiking irregularity was measured for each spike using the coefficient of variation (CV) of five successive interspike intervals (ISI), where the standard deviation (S.D.) of the ISI was divided by the mean of the ISI (CV = S.D. (ISI)/mean (ISI)). The irregularity index of each neuron was defined as the median of the CV of all of its spikes recorded during the performance of correct trials. The firing rates of tonic excitatory and suppressive neurons during the pre- and post-fixation periods were compared using the Wilcoxon rank-sum test (*p* < 0.05/6, Bonferroni correction).

## RESULTS

### INCREASE AND DECREASE IN THE TONIC ACTIVITY OF PPTN NEURONS

We analyzed the activity of neurons recorded from the PPTN while the monkeys performed a reward-biased visually guided saccade task (**Figure 1**). During fixation on the reward-conditioned FT, the monkeys could expect either a large or small reward upon the successful completion of the trial depending on the shape of the FT. Animal behavior is influenced by the predicted reward magnitude, such that there is a higher success rate and shorter saccadic reaction time to the ST in large reward-predicted trials than in those with a small reward (Okada et al., 2009).

**FIGURE 2:**
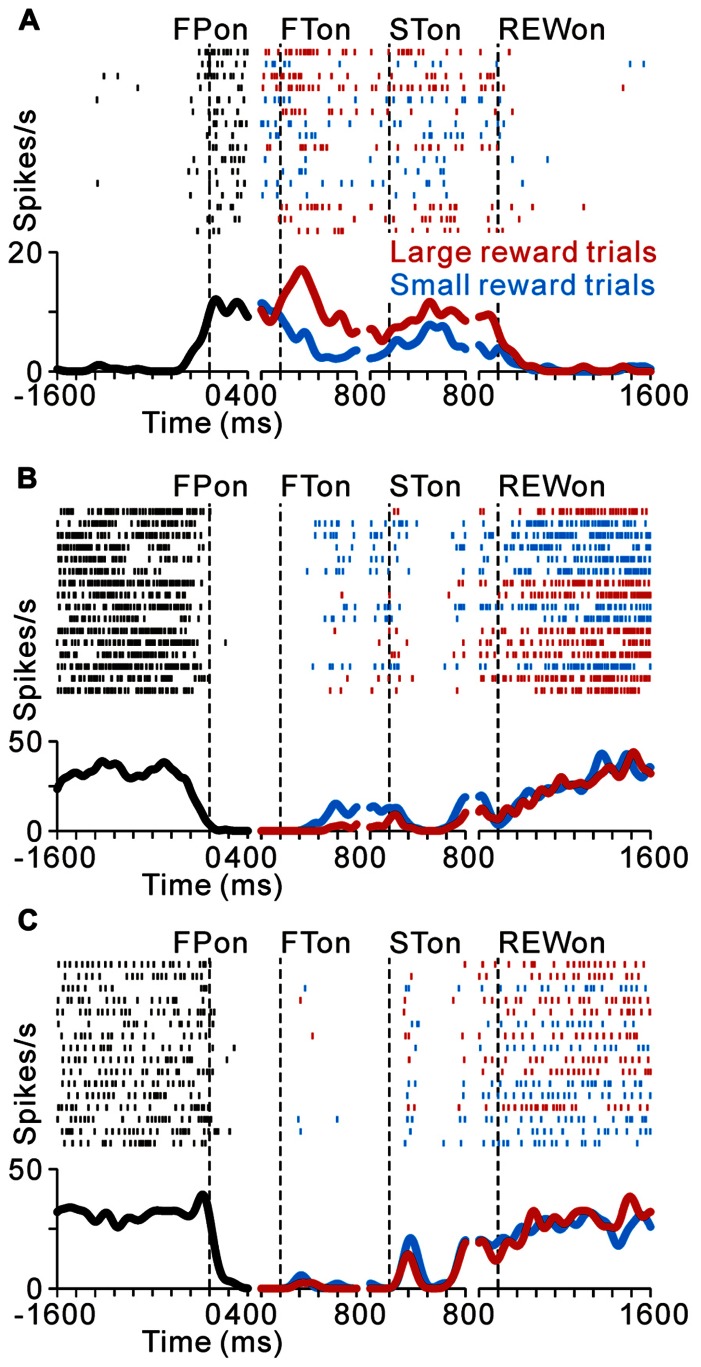
**Reward-related activity modulation of the tonic excitatory and suppressive neurons**. The rastergrams and spike density functions are shown in four sections. From left to right, the data are aligned to the time of FP onset, FT onset, ST onset, and reward delivery, respectively. The colored rasters and traces indicate the large reward trials (red), small reward trials (cyan), and all trials (black). **(A)** This representative neuron exhibited an increase in tonic activity during the task period, and showed greater activity for the large reward-predicted cue than for the small reward-predicted cue. **(B)** The activity of this representative neuron decreased during the task period, and showed smaller activity for the large reward-predicted cue. **(C)** This representative neuron also showed a decrease in tonic activity during the task period, but showed no reward prediction-related activity modulation.

As we reported previously, many PPTN neurons increase their tonic activity around the time of the initial target appearance, which was sustained until the end of the trial (tonic excitatory neurons), and some of these neurons show predicted reward-related activity modulation (Okada et al., 2009). **Figure 2A** illustrates a raster and spike density function for a representative tonic excitatory neuron. This neuron showed an anticipatory increase in activity around the time of the initial target appearance. After the reward-conditioned FT was presented during fixation, the neuron exhibited higher activity in response to the large reward-indicating FT than the small reward-indicating FT (positive reward modulation). This tonic activity and differential response ended around the time of reward delivery, and the neuronal activity was almost unrelated to the actual magnitude of the given reward (Okada et al., 2009). Thus, this neuron possibly encoded the expectation of future rewards by increasing its activity.

Here we show another group of PPTN neurons that exhibited reverse response patterns compared to the tonic excitatory neurons. **Figure 2B** illustrates an example neuron that decreased its tonic activity during the task period. Its decrease in activity started before the appearance of the FP, and then showed lower activity to a large reward-indicating FT (negative reward modulation). This differential response faded away during the saccade period and its activity returned to the pre-fixation level after reward delivery. There was no significant difference in activity according to the magnitude of the given reward. Thus, opposite to the tonic excitatory neurons, this neuron might encode the expectation of future rewards by decreasing its activity. Some tonic suppressive neurons additionally responded to multiple task events, such as the appearance of the visual stimulus and saccade, similar to the tonic excitatory neurons (Okada and Kobayashi, 2009; Okada et al., 2009). The example neuron shown in **Figure 2C** decreased its tonic activity during the task period and showed no activity modulation with the magnitude of the predicted reward, but showed a phasic burst of activity with saccades toward the ipsilateral side.

**FIGURE 3:**
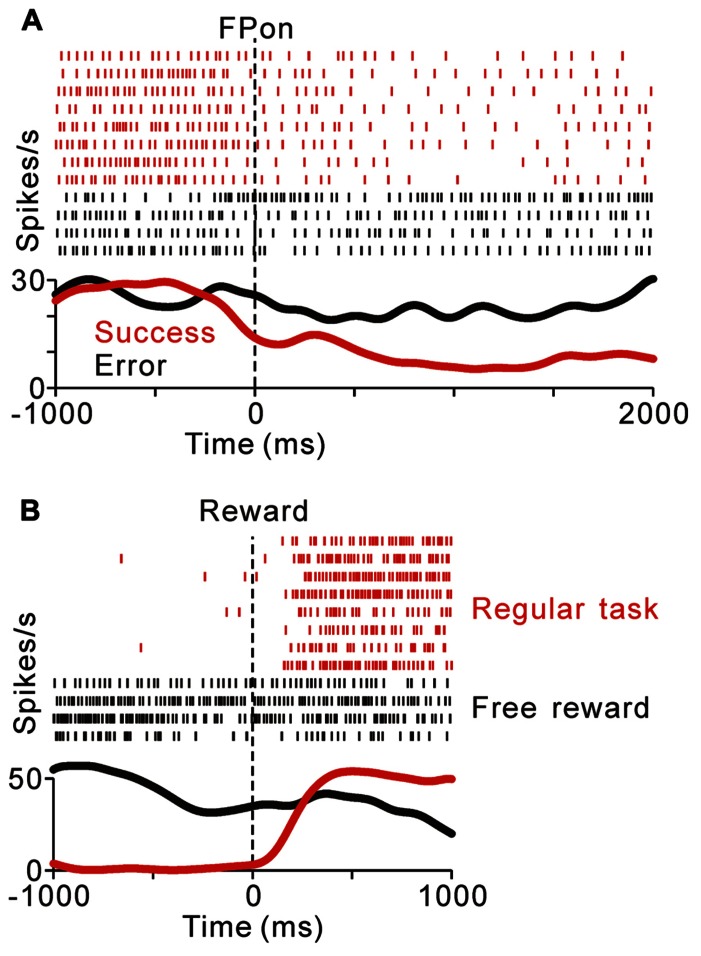
**(A)** Response of a representative tonic suppressive neuron in successful (red) and erroneous (black) trials. In the successful trials, this neuron showed a decrease in tonic activity around the time of FP appearance; however, there was no decrease in activity in the erroneous trials. **(B)** The rastergrams and spike density functions for a representative tonic suppressive neuron aligned to the delivery of free (black) and task (red) rewards. This neuron exhibited a rebound of activity shortly after reward delivery in the task condition, but remained totally unresponsive to an unexpectedly delivered reward.

Previously, we reported that the activity of tonic excitatory neurons was correlated with the monkeys’ behavioral performance, such that the neuronal responses were stronger for successful trials than for erroneous trials (Kobayashi et al., 2002; Okada et al., 2009). Tonic suppressive neurons also showed behavioral performance-related activity modulation. **Figure 3A** compares the activity of a representative tonic suppressive neuron in successful and erroneous trials, in which the monkey failed to fixate on the central target. In successful trials, this neuron showed a decrease in tonic activity around the time of FP appearance; however, there was no decrease in its activity in erroneous trials. This result suggests that tonic suppressive neurons might signal the attentional and/or motivational state, similar to the tonic excitatory neurons.

As shown in **Figures 2B,C**, tonic suppressive neurons showed an increase in tonic activity after task reward delivery that was sustained until the start of the next trial. However, there was no reward magnitude-related difference in activity after reward delivery. To examine further whether the signal of tonic suppressive neurons actually encoded reward information, we compared their responses to rewards that were delivered expectedly during the saccade task and delivered unexpectedly during the intertrial interval. **Figure 3B** shows a representative example that decreased its tonic activity during the task period and exhibited a rebound of activity shortly after reward delivery in the task condition. However, this neuron remained totally unresponsive to unexpectedly delivered rewards. This result suggests that tonic suppressive neurons do not encode the signal for the actual reward, but encode the predicted reward signal by decreasing their activity, similar to tonic excitatory neurons.

**FIGURE 4:**
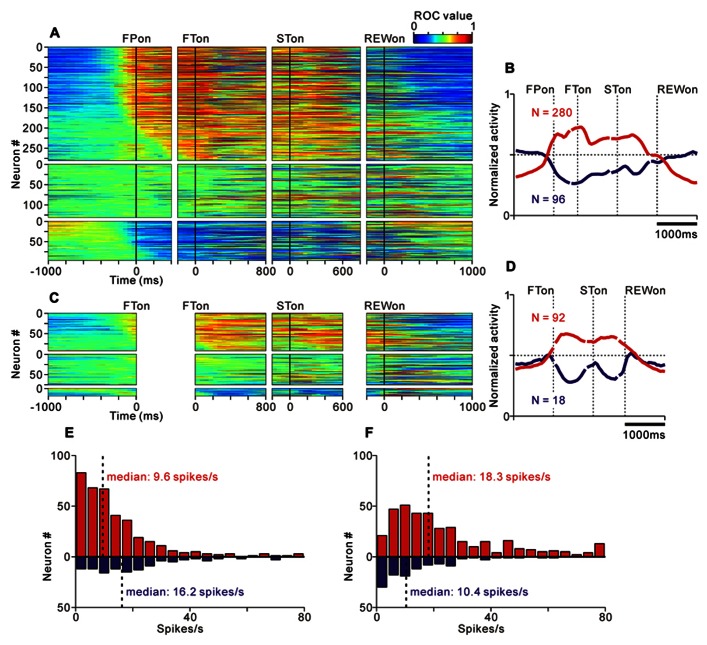
**Activity of the tonic excitatory and suppressive neurons of the PPTN for the saccade task**. **(A–C)** The activity of each PPTN neuron is presented as a row of pixels for the task with (**A**, *N* = 507) and without (**C**, *N* = 185) FP presentation. From left to right, the data are aligned to the time of FP/FT onset, FT onset, ST onset, and reward delivery, respectively. The data were plotted separately for neurons that showed an increase in tonic activity (top), no significant modulation (middle), and a decrease in tonic activity (bottom). The neurons have been sorted in the order of their initiation of changes in tonic firing. The color of each pixel indicates the ROC value based on the comparison of the firing rate between a pre-fixation period just before the appearance of the initial stimulus (600 ms duration) and a test window centered on the pixel (200 ms duration). The warm colors (ROC value >0.5) indicate increases in the firing rate relative to the pre-fixation period, whereas the cool colors (ROC value <0.5) indicate decreases in the firing rate. **(B–D)** Population average activity is shown for the task with **(B)** and without **(D)** FP presentation, separately for tonic excitatory neurons (red) and tonic suppressive neurons (blue). **(E,F)** Histograms for the firing rate during the pre- **(E)** and post-fixation **(F)** periods for the tonic excitatory (red) and suppressive (blue) neurons.

We found that many PPTN neurons showed a task-related increase or decrease in tonic activity. As reported previously, more than half of the PPTN neurons increased their activity around the time of the initial target appearance and this activity was sustained until the end of the trial (tonic excitatory neurons, *N* = 372, 54%), regardless of whether the initial target was an uninformative FP (indicating the start of a trial, top panel of **Figure 4A** and red trace of **Figure 4B**) or a reward-conditioned FT (indicating both the start of a trial and reward magnitude, top panel of **Figure 4C** and red trace of **Figure 4D**). Furthermore, another group of PPTN neurons showed a reverse response pattern; their activity was tonically decreased around the time of the initial target appearance and rebounded at the end of the task (tonic suppressive neurons, *N* = 114, 16%, bottom panels of **Figures 4A–C** and blue trace of **Figures 4B–D**. Some of the remaining neurons exhibited various phasic discharges to the visual stimulus, saccade, and reward delivery (Okada and Kobayashi, 2009; Okada et al., 2009). Here, we focused on the neuronal data showing tonic changes in activity.

Tonic excitatory and suppressive neurons started to change their tonic activity even before the appearance of the initial target, both in the fixed and quasi-randomized intertrial interval conditions. We first compared the time course of this anticipatory modulation for tonic excitatory and suppressive neurons. The start of the changes in neuronal activity was defined as the time at which the normalized activity exceeded that of the pre-fixation period by more (or less) than two standard deviations. Many tonic excitatory neurons showed an increase in activity before the appearance of the initial target (*N* = 284, 76% of tonic excitatory neurons). Similarly, many tonic suppressive neurons also started to decrease their activity before the appearance of the initial target (*N* = 74, 65% of tonic suppressive neurons). These preparatory changes in activity were slightly more frequent in tonic excitatory neurons than in tonic suppressive neurons (*p* < 0.05, chi-square test). Thus, the tonic activity was triggered not only by the appearance of the external visual stimulus but also by the anticipation of the upcoming event and/or motivation of the monkeys. We will discuss the relationship between the preparatory changes in activity and the monkeys’ anticipatory behavior in detail in a later section.

Previous *in vitro* studies reported that the neurotransmitter of PPTN neurons might be related to their electrophysiological properties, such as spike duration, spiking irregularity, and baseline firing rate (Takakusaki et al., 1996). However, it is difficult to classify neurons by these properties in extracellular recording experiments (Matsumura et al., 1997; Kobayashi et al., 2002). We tested whether these properties were correlated with the task-related tonic activity pattern, but we found no clear evidence for such a correlation. When comparing the firing rate of tonic excitatory and suppressive neurons during the pre-fixation period (600 ms period before the appearance of the initial stimulus), the tonic excitatory neurons exhibited a significantly lower frequency firing rate than the tonic suppressive neurons (**Figure 4E**, median, 9.6 spikes/s for tonic excitatory neurons, 16.2 spikes/s for tonic suppressive neurons, *p* < 0.001). However, when comparing the firing rate during the active period (post-fixation period of the tonic excitatory neurons and pre-fixation period of the tonic suppressive neurons) and silent period (pre-fixation period of the tonic excitatory neurons and post-fixation period of the tonic suppressive neurons), there was no significant difference (*p* = 0.09 for the active period, *p* = 0.58 for the silent period). Therefore, we concluded that these two groups of neurons showed a similar range of firing rates. In addition, tonic excitatory and suppressive neurons did not show a significant difference in spike duration (median, 0.53 ms for both tonic excitatory and suppressive neurons, *p* = 0.87), spiking irregularity (median CV, 0.57 for tonic excitatory neurons and 0.51 for tonic suppressive neurons, *p* = 0.17), and recording site (data not shown, see also Okada et al., 2009).

Thus, we concluded that some PPTN neurons increased, while others decreased, their tonic activity during the task period, and had a similar time course of modulation and range of firing characteristics. Therefore, these two groups of PPTN neurons showed mirror image activity patterns.

### CORRELATION BETWEEN TASK-RELATED TONIC ACTIVITY AND REWARD-RELATED MODULATION

We then tested the hypothesis that the tonic changes in the activity of PPTN neurons encoded the state of expectation of future rewards, as DRN neurons do, by analyzing the relationship between the tonic activity during the task execution period and the differential responses to the reward cues and actual reward delivery. Even if the initial target was an uninformative FP, the start of the task could be a clue for the future reward value after the successful completion of a trial (possibly the mean value of the large and small rewards). Therefore, if the tonic activity reflected the monkeys’ expectation of future rewards, then the tonic excitatory neurons should exhibit stronger activity to large reward cues (positive reward modulation; activity increased during a positive state). Thus, the tonic suppressive neurons should exhibit weaker activity to large reward cues (negative reward modulation; activity decreased during a positive state). Conversely, if the tonic changes in activity were independent of the expectation of the reward value and encoded some variables about the behavioral task, there would be no systematic relationship between the sign of tonic activity changes and reward-related activity modulation.

**FIGURE 5:**
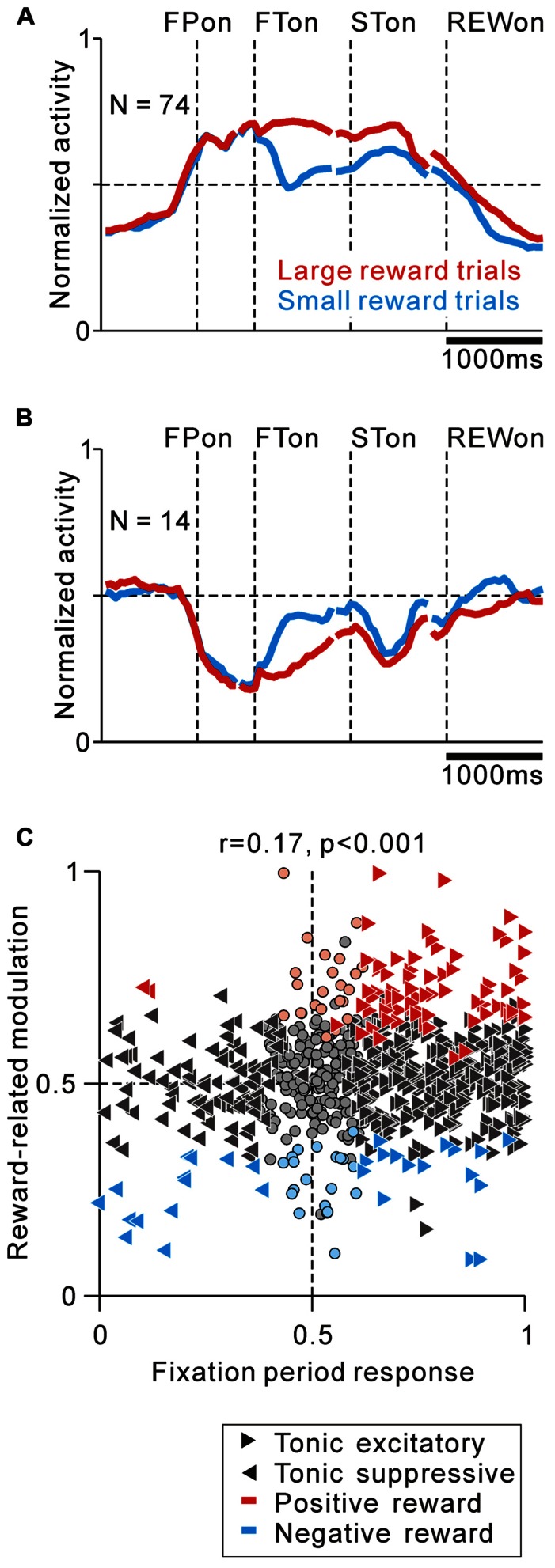
**Correlation between task-related tonic activity and reward-related modulation**. **(A,B)** Population average activity is shown separately for tonic excitatory neurons with positive reward modulation **(A)** and tonic suppressive neurons with negative reward modulation **(B)**. Neurons recorded with and without FP presentation are included. **(C)** Plot of the fixation period response (*x*-axis) versus reward-related modulation (*y*-axis). The fixation period response was measured as the ROC value for each neuron to discriminate between its firing rates during the post-fixation period (0–600 ms after the appearance of the initial stimulus) versus the pre-fixation period (0–600 ms before the appearance of the initial stimulus). Reward-related modulation was measured between its firing rates at 0–600 ms after the appearance of the reward-conditioned FT for large versus small reward trials. The marker shapes indicate neurons with a significant increase (rightward triangles) and decrease (leftward triangles) in activity during the post-fixation period (*p <* 0.05). The marker colors indicate neurons that showed significantly higher (red) and lower (cyan) activity during the large reward trials (*p <* 0.05).

As shown in **Figure 2A**, some tonic excitatory neurons exhibited higher activity in response to the large reward-indicating FT than the small reward-indicating FT. This positive reward coding was the major pattern of the predicted reward-related modulation of the tonic excitatory neurons. The population average normalized activity is shown in **Figure 5A** for neurons that showed an increase in tonic activity and positive reward modulation for the reward-conditioned FT (20% of tonic excitatory neurons, *N* = 74/372). The data from 2 reward tasks were pooled and presented together because they showed similar results. At the population level, activity modulation started even before the appearance of the initial target. If the large reward cue appeared, the higher activity was maintained, whereas if the small reward cue appeared, the activity decreased, but was still higher than the activity during the intertrial interval. Consistent with our previous finding, this differential response to the reward cue was dependent on the predicted reward magnitude rather than the shape of the FT (*p* < 0.05 and *p* > 0.1, respectively, multiple regression analysis), indicating that the tonic activity encoded the magnitude of the predicted reward rather than a simple visual response to the target stimulus (Okada et al., 2009). In a subset of the tonic excitatory neurons with positive reward modulation, the predicted reward-related differential response was maintained until shortly after reward delivery (*N* = 17/74). A small population of tonic excitatory neurons (*N* = 16/372) showed a weak negative reward modulation, in that their response was smaller for the large-reward indicating cue.

Tonic suppressive neurons also showed a correlation between their task-related tonic activity and reward-related modulation. Some tonic suppressive neurons showed negative reward modulation to the FT (13% of tonic suppressive neurons, *N* = 14/110, **Figure 5B**). Opposite to the population activity pattern of the tonic excitatory neurons, the tonic suppressive neurons keep silent during the presentation of a large reward cue, whereas if a small reward cue appeared, their activity increased, but it was still lower than their activity during the intertrial interval. Similar to the tonic excitatory neurons, the differential response to the reward cue was dependent on the predicted reward magnitude rather than the shape of the FT (*p* < 0.05 and *p* > 0.1, respectively, multiple regression analysis). Some neurons maintained negative reward modulation until shortly after reward delivery (*N* = 6/14), and only two tonic suppressive neurons showed positive reward modulation to the FT.

We then analyzed the correlation between the strength of tonic activity modulation during the task execution period and the differential response to the reward cue and reward delivery in order to examine the pattern of reward value coding in PPTN neurons. We used ROC analysis to measure the strength of tonic activity modulation and reward-related response of each neuron (Green and Swets, 1966). We found that the modulation of tonic activity during the fixation period was positively correlated with its reward-related modulation to the reward-conditioned FT (0–600 ms after FT appearance, *r* = 0.17, *p* < 0.001, **Figure 5C**), but not after reward delivery (0–600 ms after reward delivery, *r* = 0.04, *p* = 0.14, data not shown). This result further supported the view that PPTN neurons encoded the prediction of future rewards by their bi-directional changes in tonic activity, but did not primarily encode the actual reward value information. These tonic firing PPTN neurons might play a role in the reward prediction-based behavioral control system rather than the actual reward-based feedback valuation system.

Then, we examined the relationship between the absolute strength of tonic activity modulation during the task execution period and the absolute strength of the reward-related modulation after FT presentation. We used the absolute value of the difference between the ROC value and 0.5. The absolute ROC value for tonic activity modulation was not correlated with the absolute ROC value for reward-related modulation (*r* = 0.02, *p* = 0.34), possibly because there was a substantial number of neurons that showed strong tonic changes in activity during the task period, but had no reward-related modulation. Overall, we found no relationship between the absolute strength of the tonic activity during the task and the absolute strength of the reward-related modulation; however, the sign of the reward-related modulation could be predicted by the increase or decrease in tonic activity.

Thus, we concluded that some PPTN neurons encoded the tonic reward value prediction signal either by increasing or decreasing their firing rate.

### CORRELATION BETWEEN TASK-RELATED TONIC ACTIVITY AND ANTICIPATORY BEHAVIOR-RELATED MODULATION

We then examined whether the tonic changes in the activity of PPTN neurons are correlated with the anticipatory behavior of the monkeys. Previously, we reported that the predictive increase in activity before the task period was correlated with the monkeys’ anticipatory behavior, which possibly reflected the monkeys’ prediction of an upcoming visual event and motivational state (Okada et al., 2009). Our monkeys often made an anticipatory gaze shift, i.e., a self-initiated movement based on their anticipatory preparation for an upcoming visual event. The RTit was determined as a measure of the monkeys’ behavior and, possibly, their state of anticipation of an upcoming event. We classified the trials according to the RTit into short and long RTit categories (shorter and longer RTit than the median values of the individual neuron, respectively) and compared neuronal activity just before the start of the task (0–600 ms before the appearance of the initial stimulus).

**FIGURE 6:**
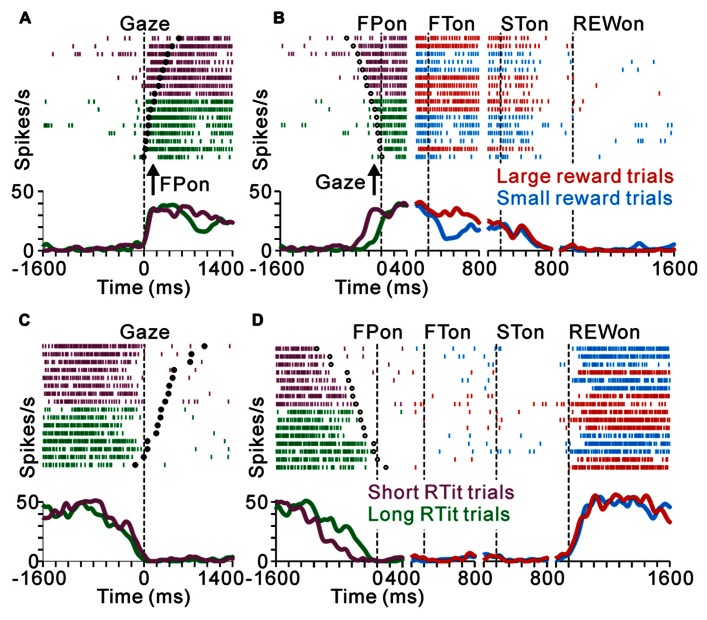
**Anticipatory behavior-related activity modulation of the tonic excitatory and suppressive neurons**. Examples of neuronal activity during the same set of trials are shown. The rastergrams and spike density functions are aligned to the monkeys’ gaze shift to the center of the screen **(A–C)** and initial target appearance **(B–D)**. The trials are sorted by RTit. The filled and open circles indicate initial target appearance and gaze shift, respectively. The colored rasters and traces indicate the short RTit trials (purple), long RTit trials (green), large reward trials (red), and small reward trials (cyan). **(A,B)** This representative neuron exhibited an increase in tonic activity during the task period, and the increase in activity started before the appearance of the FP in a behavior-dependent manner. **(C,D)** The activity of this representative neuron was decreased in a time-locked manner to the monkey’s centering gaze shift.

The anticipatory response was correlated to the monkeys’ anticipatory behavior. **Figures 6A,B** illustrates the activity of a representative tonic excitatory neuron during the same set of trials aligned to the gaze shift to the center of the screen (**Figure 6A**) and appearance of the FP (**Figure 6B**). This neuron showed a predictive increase in activity before FP appearance, and this tonic activity persisted during the task period. The start of the changes in neuronal activity was time locked to the gaze shift to the center of the screen (**Figure 6A**), rather than the appearance of the FP (**Figure 6B**). In other words, the neuron showed higher anticipatory activity in the short RTit trials than in the long RTit trials (positive behavioral modulation, **Figure 6B**). Activity reached a plateau shortly after the anticipatory gaze shift, even before the appearance of the FP. After the reward-conditioned FT was presented during fixation, this neuron also showed higher activity in response to the large reward-indicating FT than the small reward-indicating FT (positive reward modulation).

Similarly, tonic suppressive neurons showed a predictive decrease in activity before the appearance of the initial stimulus in an RTit-dependent manner. **Figures 6C,D** shows a neuron that decreased its tonic activity during the task period. Similar to the tonic excitatory neuron shown in **Figures 6A,B**, the pause in its activity was started at the time of the centering gaze shift (**Figure 6C**). Therefore, this neuron showed higher anticipatory activity in the long RTit trials than in the short RTit trials (negative behavioral modulation), and this differential response was only apparent in the pre-fixation period (**Figure 6D**).

**FIGURE 7:**
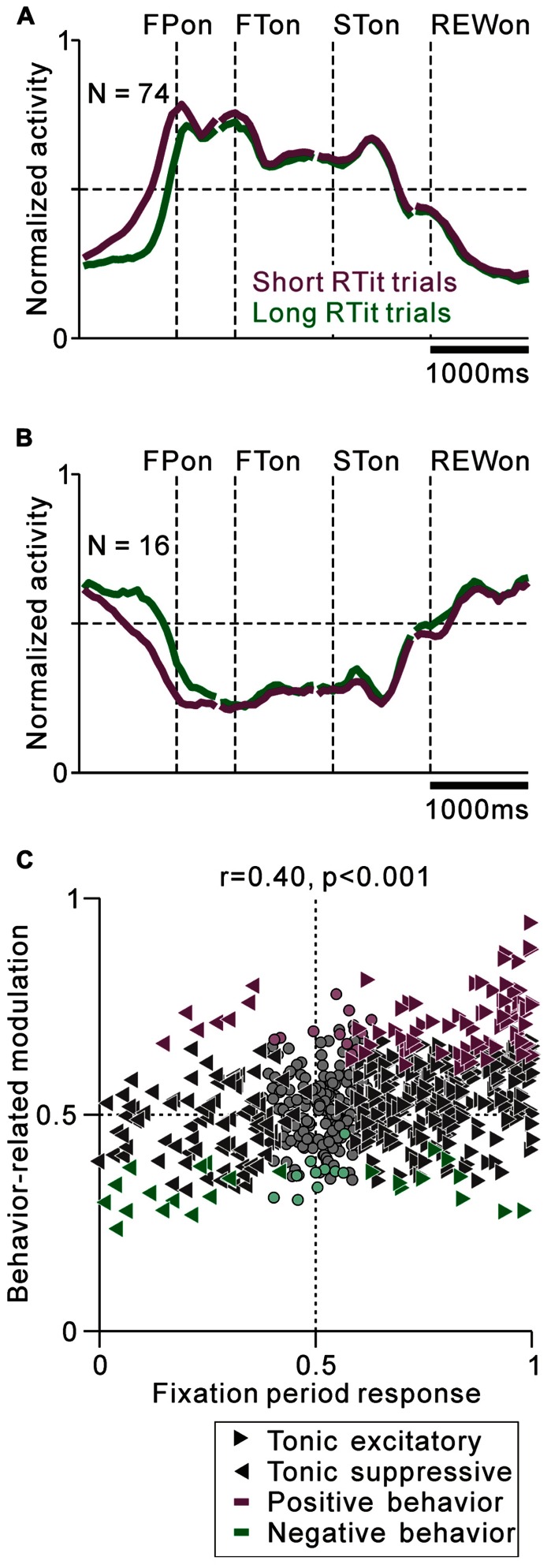
**Correlation between task-related tonic activity and behavior-related modulation**. **(A,B)** Population average activity is shown separately for the tonic excitatory neurons with positive behavioral modulation **(A)** and tonic suppressive neurons with negative behavioral modulation **(B)**. **(C)** Plot of the post-fixation period response (*x*-axis) versus behavior-related modulation (*y*-axis). The post-fixation period response was measured as the ROC value for each neuron to discriminate between its firing rates at 0–600 ms after the appearance of the initial stimulus versus the pre-fixation period at 0–600 ms before the appearance of the initial stimulus. Behavior-related modulation was measured between its firing rates at 0–600 ms before the appearance of the initial target for short versus long RTit trials. The marker shapes indicate neurons with a significant increase (rightward triangles) and decrease (leftward triangles) in activity during the post-fixation period (*p <* 0.05). The marker colors indicate neurons showing significantly higher (purple) and lower (green) activity during the short RTit trials (*p* < 0.05).

We found correlations between the tonic activity modulation and anticipatory behavior-related modulation. The population average normalized activity is shown in **Figure 7A** for neurons that showed an increase in tonic activity and positive behavioral modulation. If the monkeys made a short RTit for a centering gaze shift, a subset of tonic excitatory neurons showed higher activity during the pre-fixation period and this activity reached a plateau before the appearance of the FP, whereas if the monkeys made a long RTit for a centering gaze shift, there was a slower increase in activity (20% of tonic excitatory neurons, *N* = 74/372, **Figure 7A**). There was a small population of tonic excitatory neurons (*N* = 10) that showed a weak negative behavioral modulation in that their response was smaller in the shorter RTit trials. Opposite to the tonic excitatory neurons, some tonic suppressive neurons showed higher activity during the pre-fixation period in the short RTit trials than in the long RTit trials (14% of tonic suppressive neurons, *N* = 16/110, **Figure 7B**), and only seven neurons showed positive behavioral modulation. Thus, the increase or decrease in tonic activity reflected the motivational and/or attentional state of the monkey based on the anticipation of an upcoming event.

We then analyzed the correlation between the strength of tonic activity modulation and anticipatory behavior-related modulation. We found that the strength of tonic activity modulation during the task execution period was positively correlated with anticipatory behavior-related modulation (0–600 ms before initial stimulus appearance, *r* = 0.40, *p* < 0.001, **Figure 7C**). Furthermore, the absolute strength of the anticipatory response could be predicted by the tonic activity modulation during the task. The absolute ROC value for the tonic activity modulation was positively correlated with the absolute ROC value for the anticipatory response (*r* = 0.28, *p* < 0.01). Thus, the sign and strength of the anticipatory behavior-related modulation could be predicted by the increase or decrease in tonic activity. Neurons that had behavioral dependency basically showed a predictive change in their firing rate. We also analyzed the effect of reward history on the monkeys’ behavior and neuronal activity, but there was no significant correlation.

**FIGURE 8:**
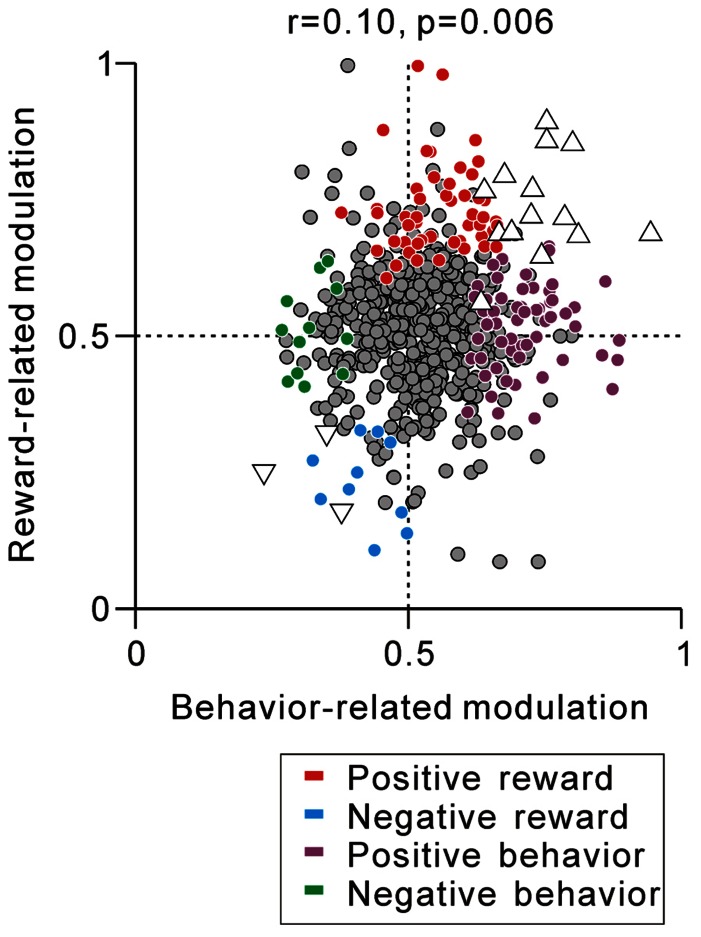
**Correlation between reward- and behavior-related modulation**. A plot of behavior-related modulation (*x*-axis) versus reward-related modulation (*y*-axis) is shown. Behavior-related modulation was measured between its firing rates at 0–600 ms before the appearance of the initial stimulus for short versus long RTit trials. Reward-related modulation was measured between its firing rates at 0–600 ms after the onset of the reward-conditioned FT for large versus small reward trials. The marker colors indicate neurons showing positive reward modulation (red), negative reward modulation (cyan), positive behavioral modulation (purple), and negative behavioral modulation (green) (*p <* 0.05). The white triangles indicate neurons showing correlated reward- and behavior-modulation by an increase (upward triangles) and decrease (downward triangles) in activity (*p <* 0.05).

Thus far, we have described separately the correlation between the tonic activity modulation during the task and predicted reward value-related modulation (**Figure 5C**) and anticipatory behavior-related modulation (**Figure 7C**). We then questioned whether PPTN neurons encoded the externally cued predicted reward value and internal anticipatory state by a correlated increase or decrease in their tonic neuronal activity. The example neuron in **Figure 6B** showed correlated encoding such that the neuron initially showed behavior-related anticipatory increases in activity and then showed a predicted reward-related differential response. **Figure 8** shows the correlation between the strength of reward-related modulation and behavior-related modulation. Some neurons showed both predicted reward value-related activity modulation and anticipatory behavior-related activity modulation (triangles, *N* = 14 for tonic excitatory neurons and *N* = 3 for tonic suppressive neurons). In addition, by correlation analysis, we found that the predicted reward-related modulation after FT presentation was positively correlated with anticipatory behavior-related modulation before the appearance of the initial stimulus (*r* = 0.10, *p* = 0.006, **Figure 8**). On the other hand, largely separate groups of neurons showed either predicted reward value-related activity modulation (red, cyan) or anticipatory behavior-related activity modulation (purple, green). Thus, the prediction of the reward-value signal and anticipatory behavior-related signal converged in a subset of PPTN neurons, while other separate populations of neurons carried these two signals independently.

## DISCUSSION

We found that most PPTN neurons showed a tonic increase or decrease in activity during the task execution period, and the sign of tonic activity modulation was correlated with their response magnitude to large/small reward cues. This result suggests that the tonic activity of PPTN neurons encodes the prediction of a future reward. Additionally, the modulation of tonic activity during the task was also correlated with the monkeys’ anticipatory behavior. Thus, the tonic activity of PPTN neurons also reflects the monkeys’ motivational and/or attentional state, which was based on the anticipation of an upcoming event. Altogether, some PPTN neurons increased, while others decreased, their activity driven either by the externally cued reward value or internal anticipatory state. These bi-directional modulation patterns with reward and motivation are in agreement with the presumed role of the PPTN in reward processing and motivational control.

Some previous studies reported the phasic activity of PPTN neurons in response to a given reward (Dormont et al., 1998; Kobayashi et al., 2002; Okada et al., 2009; Norton et al., 2011). Previously, we reported that individual different PPTN neurons showed task-related tonic activity and actual reward-related phasic response (Okada et al., 2009). Consistent with this, we found that the modulation of tonic activity during the fixation period was not correlated with the actual reward magnitude-related activity modulation after reward delivery. Another group of neurons, i.e., the tonic suppressive neurons we reported here, showed an increase in tonic activity after task reward delivery that was sustained until the start of the next trial. However, the rebound in activity was not primarily the reward magnitude-related response because: (1) the strength of rebound activity did not change with the actual reward magnitude and (2) tonic suppressive neurons remained totally unresponsive to unexpectedly delivered rewards. Therefore, tonic excitatory and suppressive neurons encode the predicted reward value by increasing and decreasing their tonic activity, and further, a separate population of PPTN neurons encode the actual reward value by a phasic increase in their activity (Okada et al., 2009).

Because obtaining a reward and avoiding a punishment are basic desires of all animals, similar modulation of reward prediction-related neuronal activity has been reported in many brain areas, including the cerebral cortices and basal ganglia nuclei (Leon and Shadlen, 1999; Roesch and Olson, 2003; Samejima et al., 2005; Belova et al., 2008; Joshua et al., 2009; Vickery et al., 2011; Tachibana and Hikosaka, 2012). Midbrain dopaminergic neurons encode the error between reward prediction and the actual reward and act as a teacher to revise the reward prediction to match an uncertain environment and acquire the maximum reward (Schultz, 1998; Bromberg-Martin et al., 2010b). The PPTN receives signals from these reward-related structures and provides strong excitatory inputs to dopaminergic neurons (Mena-Segovia et al., 2008b; Winn, 2008). Computational models of dopaminergic neuronal firing presumed the necessity of tonic excitatory and inhibitory reward prediction signals into dopaminergic neurons to produce the reward prediction error signal (Houk et al., 1995; Montague et al., 1996). The mirror image activity patterns of reward prediction-related tonic excitatory and suppressive PPTN neurons would match the requirements of this model. Thus, PPTN neurons could send both positive and negative reward prediction components to dopaminergic neurons, which are necessary for the computation of the reward prediction error signal.

In addition to the reward prediction-related activity modulation, a somewhat overlapping group of PPTN neurons showed anticipatory behavior-related activity modulation. These neurons showed an anticipatory increase/decrease of tonic activity before the appearance of the initial stimulus that was maintained until the end of the task. Furthermore, a tonic change in neuronal activity was almost absent in the error trials. Indeed, many previous studies reported that the PPTN is involved in the motivational control system. The cholinergic projections from the PPTN to the thalamus are considered as a part of the ascending reticular activating system and have a role in motivational control (Steriade, 1996). Several motivated behaviors of rats are controlled by the PPTN (Kozak et al., 2005; Wilson et al., 2009). In conditioned cats, reversible blockage of the PPTN by the injection of muscimol caused an elongation of intertrial intervals in a lever-release task (Conde et al., 1998). The correlation between the tonic activity and the monkeys’ anticipatory behavior suggests that the tonic activity of PPTN neurons might reflect the motivational and/or attentional state of the monkey and could act as the motivational drive to start and successfully complete a behavior.

We found that somewhat overlapping, but largely separate, groups of neurons showed predicted reward value-related activity modulation or behavior-related activity modulation with bi-directional changes in tonic activity. Previous studies hypothesized functional differences between the anterior and posterior PPTN, such that the posterior PPTN is connected with the sensorimotor structure and the ventral tegmental area, whereas the anterior PPTN is connected with the forebrain and the substantia nigra pars compacta (Winn, 2008). One possibility is that the neurons that showed reward-related activity modulation belong to the anterior PPTN and play a role in reward processing, whereas the neurons that showed behavior-related activity modulation belong to the posterior PPTN and play a role in motivational control. However, we found no difference in the recording sites between the reward- and behavior-related neurons. The PPTN is also hypothesized to be an integrative interface for multimodal signals (Inglis and Winn, 1995). Therefore, another possibility is that reward- and motivation-related signals converge at the PPTN neurons, and thus the PPTN neurons encoded the externally cued predicted reward value and internally driven anticipatory behavior by a correlated increase or decrease in their tonic activity.

The PPTN is connected with other neuromodulator systems that are involved in motivated behavior. The cholinergic PPTN has reciprocal connections with the serotonergic DRN (Steininger et al., 1992; Honda and Semba, 1994), and their mutual functions reportedly control wake/sleep and locomotion (Kayama and Koyama, 2003; Takakusaki et al., 2004; Harris-Warrick, 2011). In a motivated behavioral task, DRN neurons carry state value signals that track progress through a task both before and after reward delivery (Bromberg-Martin et al., 2010a). The tonic response patterns of PPTN and DRN neurons were very similar during the task execution period during which the monkeys predict the future reward. There were also differences in their activity patterns, such that most DRN neurons continually encode the reward signal after reward delivery; however, the firing of many PPTN neurons returns to the baseline state around the time of reward delivery. These different neuromodulator systems might play a role in motivated behavioral control in parallel and interact with one another.

While the PPTN is the major source of cholinergic projections in the brainstem, it also contains glutamatergic, GABAergic, dopaminergic, and noradrenergic neurons. One simple hypothesis is that these neurochemical types of neurons correspond to the different response types such as the increase and decrease in tonic activity. For example, during a behavioral task, cholinergic/glutamatergic tonic excitatory neurons are activated, while GABAergic tonic suppressive neurons disinhibit target neurons that form a push-pull circuit and could effectively activate target neurons. However, there are no reliable electrophysiological criteria (e.g., firing rate, spike shape, and spiking regularity) to identify the neurotransmitter of the recorded neuron. Additionally, we found that the tonic activity pattern of our recorded PPTN neurons had no clear relationship with several electrophysiological properties. One future direction is to determine their neuronal activity and neurotransmitter content by using new techniques (Mena-Segovia et al., 2008a; Boucetta and Jones, 2009; Cohen et al., 2012).

In addition to the tonic excitatory neurons and their positive reward- and behavioral-modulation we reported previously (Okada et al., 2009), here, we demonstrated that other PPTN neurons showed a decrease in tonic activity during the task period. Furthermore, some of these neurons showed negative activity modulation related to the predicted reward value and anticipatory behavior. The negative reward prediction signal of the PPTN would match the requirements of the reinforcement learning model. Conversely, the role of the negative anticipatory behavior-related signal remains rather elusive. The cholinergic, serotonergic, and dopaminergic neuromodulatory systems control many brain functions in a mutually interacting manner, and an understanding of the role of each neuromodulator system in reinforcement learning and motivational behavioral control will be an important direction for future research.

## Conflict of Interest Statement

The authors declare that the research was conducted in the absence of any commercial or financial relationships that could be construed as a potential conflict of interest.
